# Neurobiological and Neuroimmune Mechanisms Linking Chronic Pain, Sleep Disturbances and Mental Health Disorders

**DOI:** 10.3390/ijms27177852

**Published:** 2026-09-02

**Authors:** Boris Burnjakovic, Harrison Moy, Aleksandar Sic, Nebojsa Nick Knezevic

**Affiliations:** 1 Department of Anesthesiology, Advocate Illinois Masonic Medical Center, Chicago, IL 60657, USA; boris.burnjakovic@gmail.com (B.B.); h_moy0812@email.campbell.edu (H.M.); aca.smed01@gmail.com (A.S.); 2 Department of Internal Medicine, Valley Health System, Las Vegas, NV 89119, USA; 3 Department of Anesthesiology, University of Illinois, Chicago, IL 60612, USA; 4 Department of Surgery, University of Illinois, Chicago, IL 60612, USA

**Keywords:** chronic pain, sleep disturbances, depression, anxiety, neuroinflammation, central sensitization, HPA axis, monoaminergic systems, cytokines, neuroimmune interactions

## Abstract

Chronic pain, sleep disturbances, and mental health disorders such as anxiety and depression disorders frequently co-occur, forming a self-reinforcing cycle that impairs daily functioning and quality of life. Chronic pain is driven by peripheral and central sensitization, the latter sustained by reciprocal microglial–astrocytic crosstalk and maladaptive neuroplasticity. Poor sleep amplifies pain through inflammation and circadian disruption. Imbalances in serotonin, dopamine, and norepinephrine, together with limbic alterations and HPA axis dysregulation, contribute to comorbid anxiety and depression. Elevated pro-inflammatory cytokines (IL-1β, IL-6, IL-8, TNF-α), NF-κB-driven neuroinflammation, and mitochondrial oxidative stress serve as key molecular links. Building on previous evidence, this review presents an updated triadic, mechanism-based framework describing the reciprocal reinforcement among chronic pain, sleep disturbances, and anxiety and depressive disorders. Consequently, therapeutic strategies targeting inflammatory cytokines, microglial and astrocytic activation, neurotransmitter imbalance, and psychological dysfunction may help address these shared neuroimmune and neuroplastic mechanisms underlying these interconnected disorders.

## 1. Introduction

Sleep disturbances, chronic pain, and mental health comorbidities such as anxiety and depression are highly prevalent conditions that frequently co-occur and interact in a bidirectional manner. These disorders form a complex clinical constellation that substantially impairs physical functioning, emotional well-being, and overall quality of life [1,2,3,4].

Chronic pain is a major public health concern in the United States, with profound consequences for daily functioning and healthcare utilization. Nationally representative data indicate that approximately 50.2 million adults, corresponding to 20.5% of the U.S. population, experience pain on most days or every day and are significantly more likely to report activity limitations and increased work absenteeism compared with individuals without chronic pain [5,6]. Chronic pain also shows marked sex-related differences in prevalence, with women generally experiencing a greater burden than men; an international survey across 17 countries reported chronic pain in 45% of women compared with 31% of men [5].

Central sensitization, a key conceptual framework underlying this review and a fundamental mechanism driving the persistence and amplification of chronic pain, is defined as an increased responsiveness of nociceptive neurons within the central nervous system to normal or subthreshold afferent input, resulting in pain hypersensitivity and sustained pain states [7].

Mental health disorders commonly coexist with chronic pain and anxiety disorders, affecting nearly one-third of U.S. adults over their lifetime and causing substantial psychological and functional impairment [8]. Nationally representative data from 2022 further show that 18.2% of U.S. adults report anxiety symptoms and 21.4% report depressive symptoms, with prevalence increasing since 2019 and disproportionately affecting younger adults, women, individuals of lower socioeconomic status, and rural populations [9].

Sleep has been shown to be an important link between pain and mental health, playing a central role in cognitive and emotional regulation. Disruptions in sleep are strongly associated with both anxiety and depressive disorders and further exacerbate pain perception and emotional dysregulation [10,11,12].

Population-based analyses from the National Health and Nutrition Examination Survey demonstrate that sleep disorders are highly prevalent among U.S. adults, with approximately 8.4% reporting a diagnosed sleep disorder and more than one-quarter experiencing troubled sleeping, a prevalence that has increased steadily over recent decades [13].

Despite this clinical and epidemiological overlap, biological mechanisms linking chronic pain, sleep disturbances, and mental health disorders, particularly anxiety and depression, are still incompletely understood. The existing literature suggests convergence across shared cellular and molecular pathways, including neuroinflammatory signaling, glial cell activation, dysregulated neurotransmitter systems, stress-related endocrine dysfunction, and maladaptive synaptic plasticity within pain- and emotion-processing networks. Sleep disruption further modulates these processes by altering circadian regulation, immune signaling and hypothalamic–pituitary–adrenal (HPA) axis activity, which amplifies both nociceptive and affective disturbances [14,15,16,17,18].

Although substantial research has examined the relationships among chronic pain, sleep disturbances, and affective disorders, their shared and reciprocal mechanisms remain incompletely understood. Previous research has described neurobiological interactions among chronic pain, depression, and sleep disruption. However, direct evidence from studies simultaneously assessing chronic pain, objectively measured sleep disturbances, and psychiatric outcomes remains limited. Consequently, the present review integrates evidence from both multidomain and pairwise mechanistic studies to provide an updated framework incorporating neuroimmune signaling, central sensitization, neuron–glia interactions, mitochondrial dysfunction, circadian dysregulation, and affective dysfunction.

**Figure 1:**
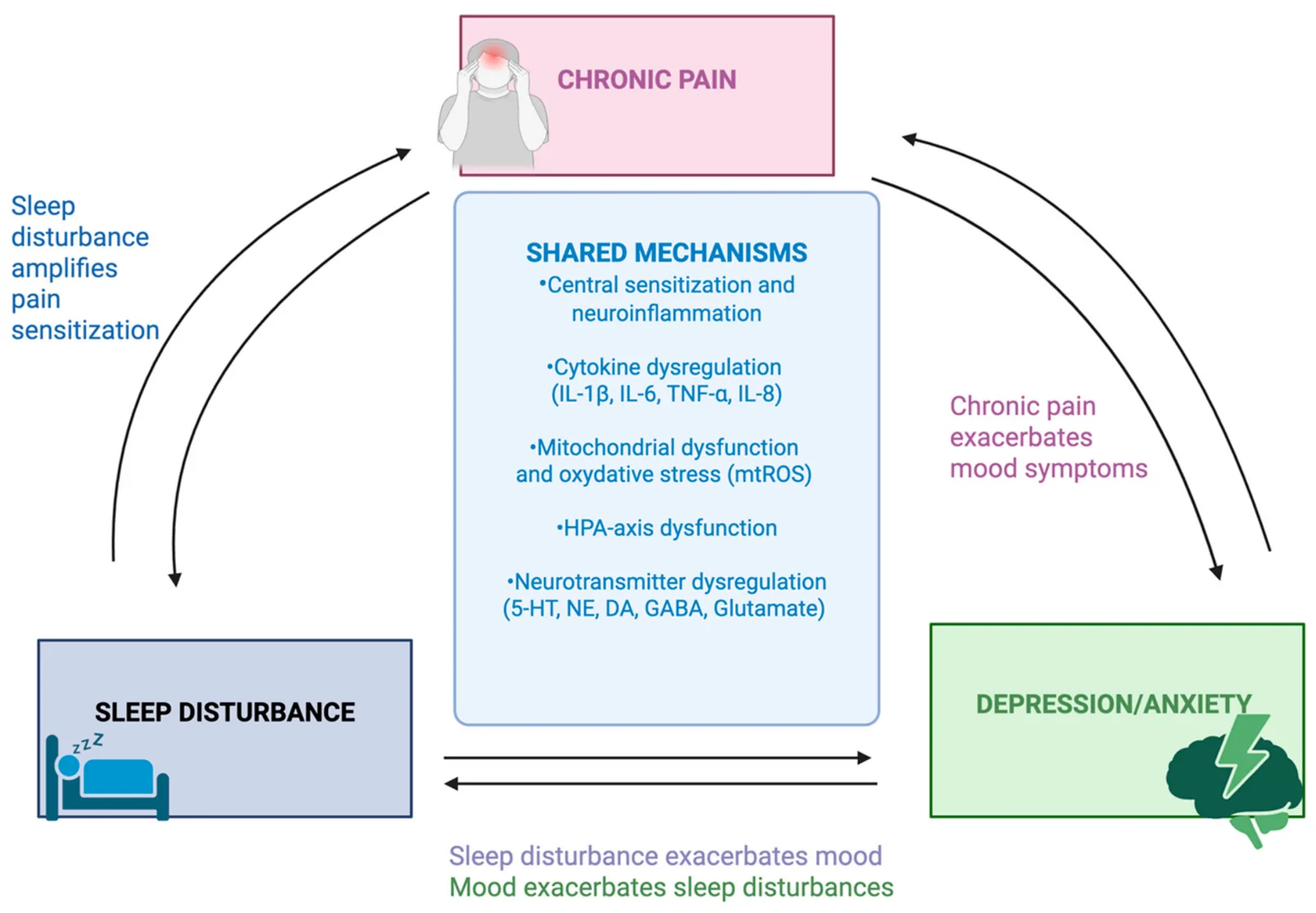
Bidirectional interactions among chronic pain, sleep disturbance, and mood disorders through shared neuroimmune, neuroendocrine, and neuroinflammatory mechanisms. IL-1β (interleukin-1 beta); IL-6 (interleukin-6); IL-8 (interleukin-8); TNF-α, tumor necrosis factor alpha; mtROS, mitochondrial reactive oxygen species; HPA axis, hypothalamic–pituitary–adrenal axis; 5-HT, serotonin (5-hydroxytryptamine); NE, norepinephrine; DA, dopamine; GABA, gamma-aminobutyric acid. Created in BioRender. Knezevic, N. (2026). https://BioRender.com/ylpxw0r.

This review proposes a triadic, mechanism-based framework comprising chronic pain, sleep disturbances, and anxiety/depression. Rather than representing a fixed linear sequence, these three domains are conceptualized as a bidirectional and self-reinforcing network in which each component can initiate or exacerbate the others (Figure 1). The initiating drivers may vary according to pain phenotype and clinical context; however, persistent nociceptive signaling, central sensitization, sleep disruption, and affective dysregulation can progressively reinforce one another. Once established, this reciprocal cycle may be maintained through convergent mechanisms, including reciprocal neuron–glia interactions, cytokine-mediated inflammation, mitochondrial dysfunction and oxidative stress, monoaminergic dysregulation, circadian disruption, and altered hypothalamic–pituitary–adrenal (HPA) axis signaling. By integrating molecular, cellular, and systems-level mechanisms, this review further highlights how these interconnected neuroimmune and neuroendocrine pathways reciprocally reinforce pain sensitization, sleep disruption, and affective dysfunction through self-perpetuating inflammatory and maladaptive neuroplastic feedback networks.

This mechanistic synthesis may improve understanding of the complex interplay between chronic pain, sleep disturbances, and anxiety and depressive disorders while also identifying potential convergent therapeutic targets and translational implications for multidisciplinary management approaches.

## 2. Literature Search Strategy

A narrative literature review was conducted to synthesize current evidence regarding the neurobiological and neuroimmune mechanisms linking chronic pain, sleep disturbances, and anxiety and depressive disorders.

The relevant literature was identified through searches of PubMed and Google Scholar databases using combinations of keywords and MeSH terms including “chronic pain”, “central sensitization”, “microglia”, “astrocytes”, “depression”, “anxiety”, “mental health”, “sleep disturbances”, “insomnia”, “neuroinflammation”, “cytokines”, “HPA axis”, “monoaminergic systems”, and “mitochondrial dysfunction”.

**Inclusion Criteria**

- Peer-reviewed original research (experimental and clinical) and reviews (systematic and narrative) evaluating neurobiological and neuroimmune mechanisms linking chronic pain, sleep disturbances, and anxiety and depression disorders.
- Studies investigating central sensitization, glial activation (microglia and astrocytes), neuroinflammatory cytokine signaling, HPA axis dysregulation, monoaminergic pathways, or circadian/melatonin disruption.
- Studies evaluating behavioral, pharmacological, or neuroimmune-targeted therapeutic strategies for these comorbid conditions.
- Studies published between 2015 and 2026, alongside seminal older literature providing foundational concepts.

**Exclusion Criteria**

- Non-peer-reviewed publications, conference abstracts, editorials, book reviews, or gray literature.
- Studies focused purely on mechanical, structural, or surgical pain interventions without investigating central, neuroimmune, or neuroendocrine mechanisms.
- Studies with limited relevance to the neurobiological, neuroimmune, or neuroendocrine mechanisms underlying chronic pain, sleep disturbances, anxiety, and depression.
- Non-English publications or studies without accessible full text.

**Figure 2:**
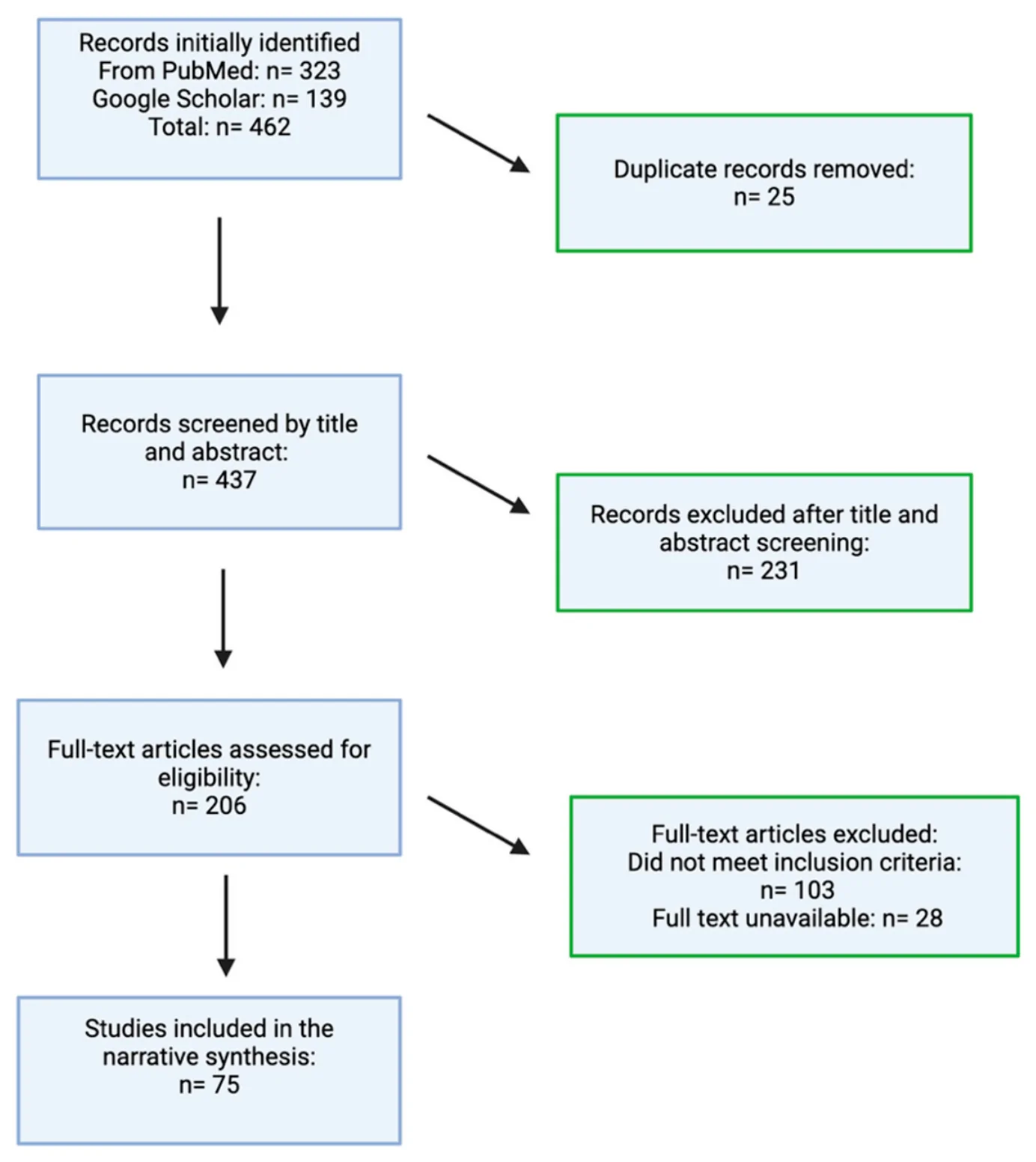
Flowchart of the literature search and study selection process. Following screening and eligibility assessment, 75 studies were included in the final narrative synthesis. Created in BioRender. Knezevic, N. (2026). https://BioRender.com/dvfcw74.

Priority was given to peer-reviewed studies published within the last 10 years (2015–2026), including experimental studies, clinical studies, systematic reviews, and narrative reviews focusing on the molecular, cellular, neuroimmune, and neuroendocrine mechanisms underlying the interactions among chronic pain, sleep disturbances, and affective disorders (Figure 2). Seminal older studies were also included when necessary to provide foundational evidence for key mechanistic concepts discussed in this review. The focus on the recent literature was intended to ensure inclusion of the most current evidence regarding neuroimmune signaling, glial activation, central sensitization, circadian regulation, and emerging therapeutic strategies, while maintaining important historical context through the inclusion of landmark studies.

Given the narrative nature of this review, both original studies and relevant review articles were included. Original experimental and clinical studies were emphasized when discussing mechanistic evidence, whereas systematic and narrative reviews were primarily used to provide contextual background, summarize established knowledge, and facilitate integration of findings across different research domains.

Articles were selected based on their relevance to the mechanistic pathways and therapeutic implications discussed in this review.

## 3. An Updated Overview of Chronic Pain

Sustained peripheral and central sensitization interact dynamically to drive persistent pain hypersensitivity, manifesting as hyperalgesia and allodynia through amplified ascending nociceptive signaling and impaired descending inhibitory modulation [19].

**Figure 3:**
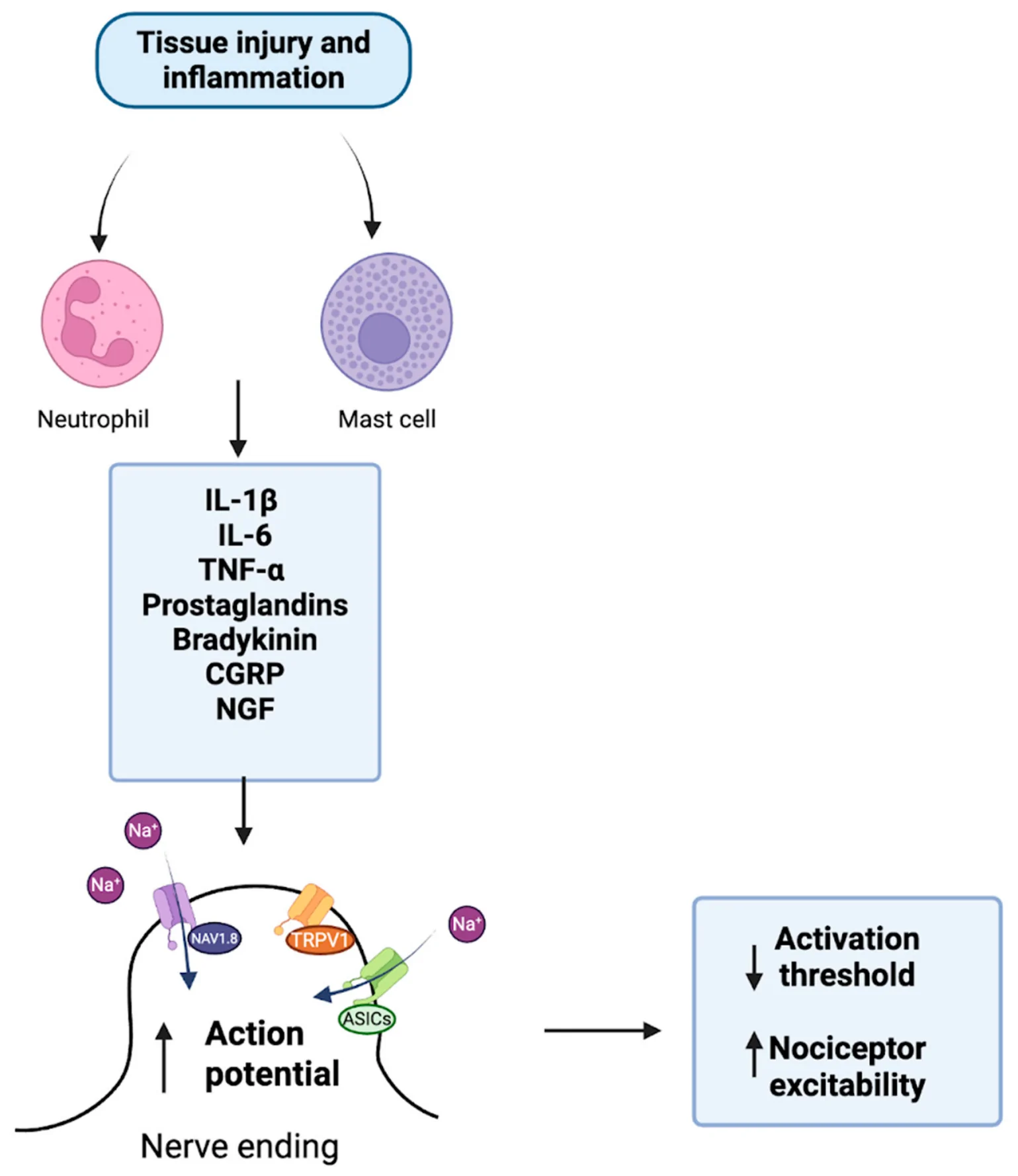
Schematic representation of peripheral sensitization following tissue injury and inflammation. Tissue injury triggers activation and recruitment of immune cells, including neutrophils and mast cells, which release a variety of pro-inflammatory mediators, such as IL-1β (interleukin-1 beta), IL-6 (interleukin-6), TNF-α (tumor necrosis factor alpha), prostaglandins, bradykinin, CGRP (calcitonin gene-related peptide), and NGF (nerve growth factor). These mediators interact with peripheral nociceptors and modulate ion channels involved in nociceptive signal transduction, including voltage-gated sodium channels (NaV1.8), transient receptor potential vanilloid 1 (TRPV1), and acid-sensing ion channels (ASICs). Activation and sensitization of these channels lower the activation threshold of nociceptors and increase neuronal excitability, resulting in enhanced action potential generation in response to noxious and, in some cases, normally innocuous stimuli. This process, known as peripheral sensitization, contributes to increased nociceptive signaling, hyperalgesia, and the development of persistent pain states. Abbreviations: IL-1β, interleukin-1 beta; IL-6, interleukin-6; TNF-α, tumor necrosis factor alpha; CGRP, calcitonin gene-related peptide; NGF, nerve growth factor; NaV1.8, voltage-gated sodium channel 1.8; TRPV1, transient receptor potential vanilloid 1; ASICs, acid-sensing ion channels. The depicted mechanisms integrate evidence from both human studies and preclinical experimental models; however, several cellular and molecular pathways remain primarily supported by preclinical evidence and require further validation in humans. Created in BioRender. Knezevic, N. (2026). https://BioRender.com/13hic40.

Peripheral sensitization is characterized by reduced activation threshold and heightened responsiveness of peripheral sensory neurons in response to inflammatory and immune mediators released at sites of tissue injury or inflammation [20]. Under these conditions, nociceptors that are normally inactive or minimally responsive become sensitized, leading to increased nociceptive input. Prostaglandins, bradykinin, tumor necrosis factor-α, interleukin-1β, and interleukin-6 interact with sensory nerve endings through transient receptor potential and voltage-gated sodium channels, lowering activation thresholds and increasing afferent firing rates (Figure 3). As a result, nociceptors respond to both noxious and previously non-noxious stimuli, amplifying peripheral signaling that is transmitted to the central nervous system and perceived as pain [19].

Central sensitization refers to an increased responsiveness of nociceptive neurons within the central nervous system to normal or subthreshold afferent input and represents a core manifestation of maladaptive CNS plasticity, characterized by heightened spontaneous neuronal activity, reduced activation thresholds, and expansion of receptive fields [7]. This phenomenon is widely observed across multiple chronic pain conditions commonly encountered in rheumatology practice, including fibromyalgia, osteoarthritis, rheumatoid arthritis, Ehlers–Danlos syndrome, headache disorders, and spinal pain [21].

**Figure 4:**
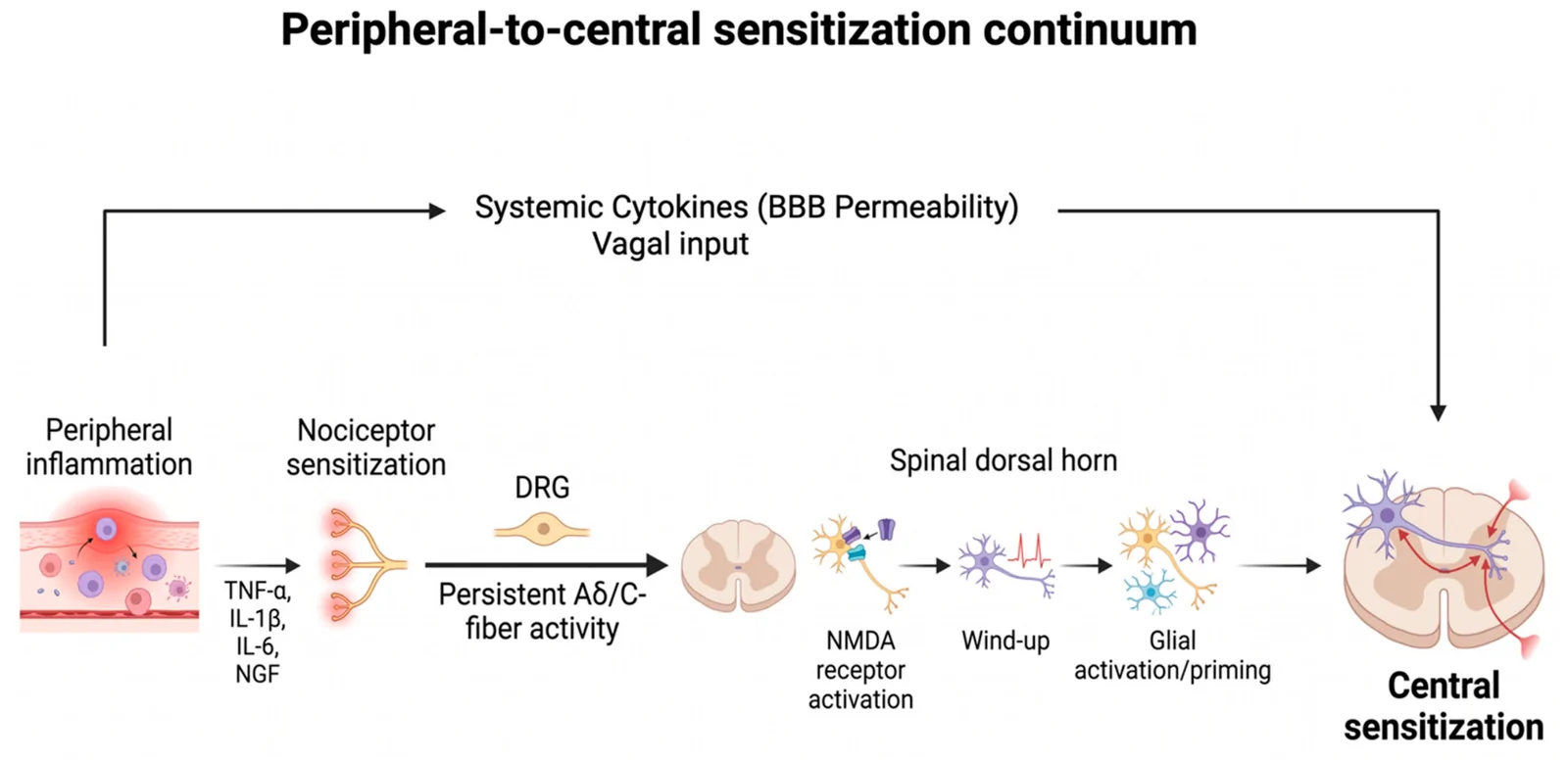
Peripheral-to-central sensitization continuum. Persistent peripheral inflammation promotes the release of inflammatory mediators, including TNF-α, IL-1β, IL-6, and NGF, leading to nociceptor sensitization and sustained Aδ- and C-fiber afferent activity transmitted through the dorsal root ganglia (DRG) to the spinal dorsal horn. Sustained nociceptive input promotes NMDA receptor activation, wind-up, and glial activation/priming, thereby facilitating the development and maintenance of central sensitization. In parallel, systemic inflammatory signals may influence central nervous system function through altered blood–brain barrier (BBB) permeability and vagal afferent pathways, providing an additional link between peripheral inflammation and central neuroimmune activation. Abbreviations: BBB, blood–brain barrier; DRG, dorsal root ganglion; IL-1β, interleukin-1 beta; IL-6, interleukin-6; NGF, nerve growth factor; NMDA, N-methyl-D-aspartate; TNF-α, tumor necrosis factor alpha. Created in BioRender. Knezevic, N. (2026) https://BioRender.com/sqgzkmi.

Central sensitization also plays an important role in the transition from acute to chronic pain by inducing a persistent hyperexcitable state within central nociceptive circuits, largely driven by glutamate-dependent activation of NMDA receptors (Figure 4). Sustained depolarization removes the magnesium block from NMDA receptors, allowing calcium influx and activation of intracellular signaling cascades that enhance synaptic efficacy and stabilize sensitized pain pathways [22,23]. Dysregulated calcium signaling has also been implicated in depression, suggesting a potential mechanistic point of convergence between maladaptive pain-related plasticity and affective dysfunction [24].

These processes enhance maladaptive neuroplastic changes, including altered membrane excitability, impaired inhibitory control, long-term synaptic modifications such as long-term potentiation and depression, and structural remodeling of axons and dendrites. These alterations then amplify nociceptive signaling beyond the site of injury, reinforce pain hypersensitivity, and underpin the development and maintenance of chronic pain [22,23].

**Figure 5:**
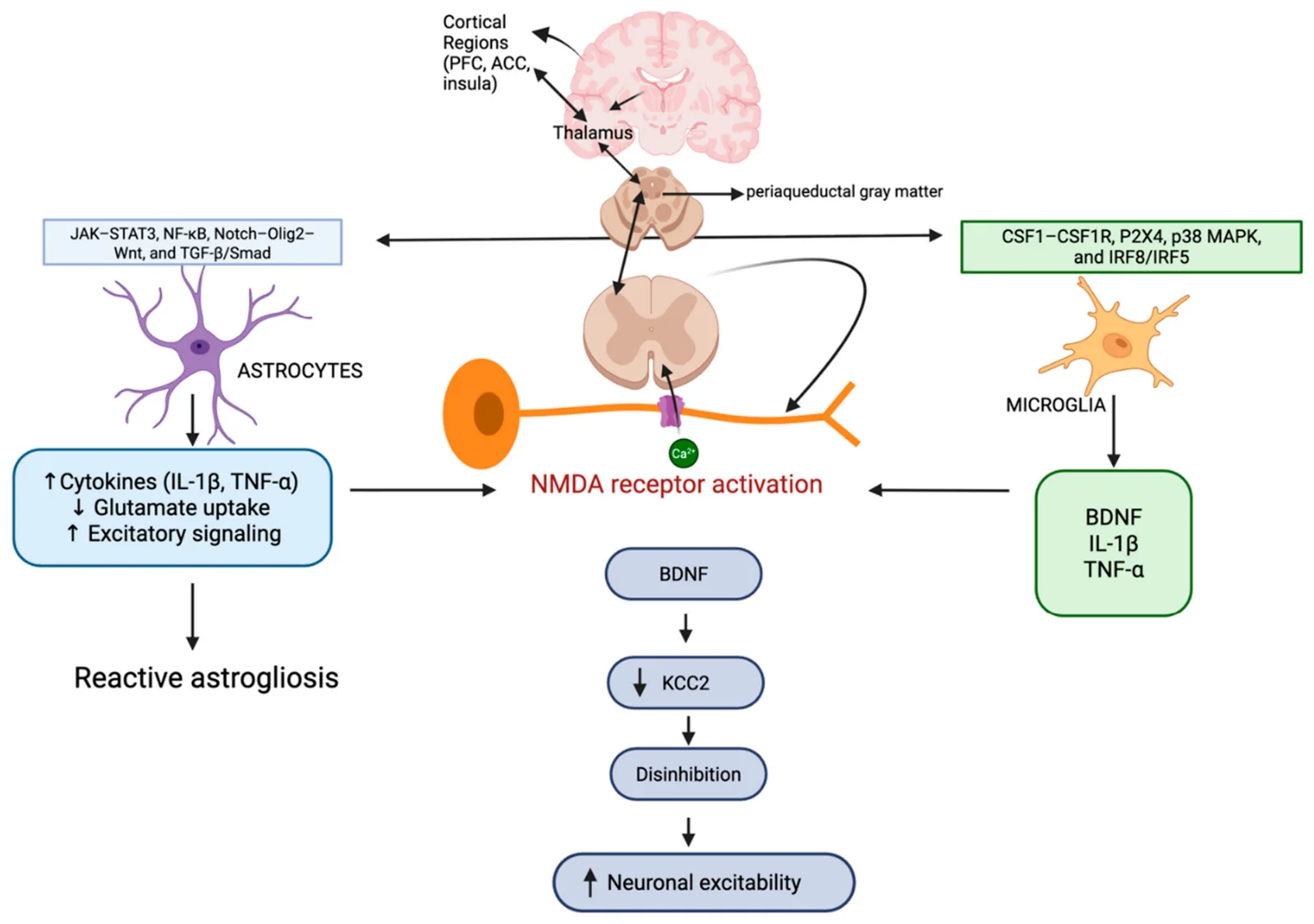
Integrated spinal and supraspinal pathways of central sensitization. Sustained nociceptive input at the spinal level triggers reciprocal astrocyte–microglia interactions. Microglial activation through CSF1–CSF1R, P2X4, p38 MAPK, and IRF8/IRF5 signaling pathways promotes the release of BDNF, IL-1β, and TNF-α, facilitating NMDA receptor activation, KCC2 downregulation, neuronal disinhibition, and enhanced dorsal horn excitability. Concurrently, reactive astrogliosis, mediated by JAK–STAT3, NF-κB, Notch–Olig2–Wnt, and TGF-β/Smad signaling, impairs glutamate clearance and amplifies pro-inflammatory signaling. Ascending nociceptive pathways relay these signals to key supraspinal structures, including the thalamus, cortical regions, and periaqueductal gray (PAG), disrupting descending inhibitory control and engaging neural circuits involved in sleep–wake regulation and emotional processing. The depicted mechanisms integrate evidence from both human studies and preclinical experimental models; however, several cellular and molecular pathways remain primarily supported by preclinical evidence and require further validation in humans. Abbreviations ACC, anterior cingulate cortex; BDNF, brain-derived neurotrophic factor; CSF1, colony-stimulating factor 1; CSF1R, colony-stimulating factor 1 receptor; IL-1β, interleukin-1 beta; IRF5, interferon regulatory factor 5; IRF8, interferon regulatory factor 8; JAK, Janus kinase; KCC2, potassium–chloride cotransporter 2; MAPK, mitogen-activated protein kinase; NF-κB, nuclear factor kappa B; NMDA, N-methyl-D-aspartate; Olig2, oligodendrocyte transcription factor 2; P2X4, P2X purinoceptor 4; PAG, periaqueductal gray; PFC, prefrontal cortex; Smad, SMAD family of signal transducers; STAT3, signal transducer and activator of transcription 3; TGF-β, transforming growth factor beta; TNF-α, tumor necrosis factor alpha; Wnt, wingless-related integration site. Created in BioRender. Knezevic, N. (2026). https://BioRender.com/qkr5mp3.

Chronic pain is also closely linked to sustained activation of microglia and astrocytes within key pain-processing regions of the central nervous system, most notably the spinal cord dorsal horn [24,25,26,27,28,29] (Figure 5).

Tissue injury triggers the release of danger-associated molecules, such as ATP and HMGB1, which, through engagement of pattern-recognition receptors, initiate early microglial activation via CSF1–CSF1R, P2X4, p38 MAPK, and IRF8/IRF5 signaling pathways, resulting in sustained release of IL-1β, TNF-α, and brain-derived neurotrophic factor (BDNF) [27,28,30]. These mediators not only enhance excitatory synaptic transmission and NMDA receptor activity but also promote reactive astrocytosis through activation of multiple intracellular signaling pathways, including NF-κB, JAK–STAT3, Notch–Olig2–Wnt, and TGF-β/Smad [31].

Importantly, preclinical evidence indicates that the contribution of spinal microglia to persistent pain hypersensitivity may differ by sex. In several rodent models, microglial inhibition or depletion reduced pain hypersensitivity predominantly in males, whereas alternative immune mechanisms, including T-cell-mediated pathways, may contribute in females; however, microglial involvement has also been demonstrated in females, indicating that these sex differences are not uniform across experimental models [32].

Consequently, astrocytes undergo transcriptional and functional reprogramming characterized by increased production of pro-inflammatory cytokines and chemokines, impaired glutamate clearance, altered metabolic homeostasis, and disruption of inhibitory neurotransmission. Activation of the gp130–JAK–STAT3 axis further promotes astrocyte proliferation within the dorsal horn and contributes to persistent tactile allodynia. Simultaneously, NF-κB signaling induces a self-amplifying transcriptional program involving IL-1β, TNF-α, CCL2, and CXCL10, thereby enhancing neuroinflammation, maladaptive synaptic plasticity, and microglial recruitment that reinforces the neuroinflammatory feedback loop underlying central sensitization [29].

In parallel, Notch-dependent Olig2 expression regulates reactive astrocyte expansion and inhibitory neurotransmission, whereas TGF-β signaling exerts a context-dependent dual role by limiting excessive glial activation while simultaneously inducing GFAP expression and Smad-dependent transcriptional changes that disrupt glutamate homeostasis and metabolic regulation [29,31,32].

The previously described reciprocal signaling between activated microglia and astrocytes establishes a self-perpetuating neuroinflammatory feedback loop that sustains central sensitization and maladaptive synaptic plasticity. Microglia-derived BDNF contributes to KCC2 downregulation and subsequent loss of inhibitory control within dorsal horn neurons, while astrocytic dysfunction promotes extracellular glutamate accumulation and diminished GABAergic inhibitory tone, collectively reinforcing neuronal hyperexcitability and pain hypersensitivity [27,28,30].

Beyond spinal mechanisms, central sensitization also involves supraspinal circuits that integrate nociceptive, affective, and behavioral responses. Ascending nociceptive signals engage the thalamus and cortical regions, including the prefrontal cortex (PFC), anterior cingulate cortex (ACC), and insula, while altered periaqueductal gray (PAG)-mediated descending modulation may further contribute to persistent pain [33,34]. Dysregulation of these interconnected supraspinal circuits may also provide a mechanistic interface through which chronic pain interacts with sleep–wake disturbances and anxiety and depression [35].

## 4. Sleep Disturbance as a Driver of Pain Sensitization

### 4.1. Neural Circuitry Linking Sleep Disturbance and Pain Sensitization

Chronic stress and persistent pain are associated with reduced sleep quality, particularly disruption of restorative N3 sleep, which is essential for tissue repair, metabolic waste clearance, immune modulation, and synaptic downscaling [36,37,38]. Experimental sleep fragmentation studies demonstrate that reduced N3 sleep is accompanied by increased IL-6 and TNF-α production and heightened pain sensitivity, suggesting that impaired slow-wave sleep promotes a pro-inflammatory state that enhances nociceptive processing and contributes to a self-perpetuating cycle between sleep disturbance and chronic pain [39].

Recent studies have identified several additional brain regions, including the nucleus accumbens, lateral hypothalamic area, ventral tegmental area, supramammillary nucleus, dorsal raphe nucleus, and locus coeruleus, as important regulators of both sleep and pain processing [40,41,42,43,44]. Notably, activation of dopamine D1 receptor–expressing neurons within specific nucleus accumbens circuits has been shown to simultaneously enhance nociceptive sensitivity and suppress non-rapid eye movement (NREM) sleep [43].

Extending this dual modulatory role, the ventral tegmental area provides major dopaminergic input coordinates pain processing and sleep–wake dynamics through coordinated glutamatergic and GABAergic neuronal activity, influencing nociceptive perception via dopaminergic projections to the medial prefrontal cortex [40].

### 4.2. Effects of Sleep Deprivation on Nociceptive Thresholds

Building upon the disruption of sleep architecture, particularly the N3 sleep stage, sleep deprivation also significantly affects nociceptive processing. These effects are especially pronounced in patients with fibromyalgia, in whom sleep deprivation consistently lowers pain thresholds and amplifies pain sensitivity. Experimental studies have demonstrated reproducible changes across multiple pain modalities [45,46,47]. Illustrating these findings, a controlled study at Aalborg University showed that 24-h total sleep deprivation in healthy participants significantly reduced cold pain threshold and cuff-induced tolerance threshold, indicating heightened pain sensitivity. Impaired conditioned pain modulation was also observed, suggesting dysfunction of descending inhibitory pain pathways. Collectively, these findings suggest that sleep deprivation enhances sensitization processes while impairing endogenous pain inhibition [45].

A blinded crossover study investigating the relationship between sleep disturbance and migraine found that partial sleep restriction modestly increased pain sensitivity in certain migraine subgroups, although overall effects did not differ significantly from those observed in healthy controls. Reduced heat and pressure pain thresholds were observed particularly in migraineurs with lower attack pain intensity, greater photophobia, or sleep-related migraine, suggesting that sleep deprivation may enhance nociceptive sensitivity in susceptible migraine populations. However, suprathreshold pain responses were not significantly affected [47].

Overall, these findings reinforce the critical role of sleep in pain regulation and suggest that targeting sleep disturbances may represent an important therapeutic strategy in the management of chronic pain.

### 4.3. Sleep Deprivation Affecting Molecular Pathways of Sleep: Circadian Rhythm Genes (CLOCK, BMAL1), Melatonin Signaling and HPA Axis Dysregulation

At the molecular level, sleep disturbances interfere with circadian regulation and neuroendocrine signaling, all of which contribute to altered pain processing [48,49,50].

Circadian rhythm regulation is governed by core clock genes, including *CLOCK*, *BMAL1*, and *PER1* which control the rhythmic expression of numerous genes involved in metabolism, immune function, and neuronal activity. Human studies demonstrate that sleep deprivation alters circadian clock gene expression. In a study of 81 participants undergoing polysomnography and total sleep deprivation, sleep loss decreased *CLOCK* and *BMAL1* expression while increasing *PER1* [51].

Disruption of these genes has been associated with increased inflammatory signaling and altered pain sensitivity. Dysregulation of circadian rhythms may therefore contribute to both heightened nociception and impaired recovery processes [48,51].

Melatonin, a key hormone regulating sleep–wake cycles, also plays an important role in pain modulation. A clinical study of patients with fibromyalgia found that disrupted daytime melatonin secretion, as measured by 24-h urinary 6-sulfatoxymelatonin (aMT6s), was significantly associated with lower pressure pain thresholds, a greater number of trigger points, and poorer sleep quality [48]. Consistent with these clinical findings, preclinical as well as human studies indicate that melatonin plays an essential role in pain modulation by coordinating the interplay between nociceptive neurons and immune cells, thereby regulating the crosstalk between inflammation and pain transmission [52]. Melatonin exerts anti-inflammatory effects through binding to MT2 receptors expressed on immune cells, inhibiting myeloperoxidase (MPO) activity and NF-κB signaling and reducing the production of pro-inflammatory mediators, including IL-1β, IL-6, and TNF-α, involved in nociceptive sensitization [52,53].

Evidence from experimental mouse and rat models further suggests that melatonin exerts additional anti-inflammatory effects by inhibiting phospholipase A2 (PLA2)-mediated arachidonic acid release and downregulating 5-lipoxygenase (5-LOX) and cyclooxygenase-2 (COX-2), thereby reducing the synthesis of chemoattractant leukotrienes and prostaglandins [54,55]. It further suppresses inducible nitric oxide synthase (iNOS) and COX-2 expression, leading to decreased production of nitric oxide (NO) and prostaglandin E_2_ (PGE_2_) [55]. Since these lipid mediators and reactive molecules are directly implicated in inflammatory pain perception, their inhibition represents a key mechanism underlying the analgesic effects of melatonin [52,53,54,55].

Melatonin also upregulates key antioxidant defense systems, including superoxide dismutase (SOD), catalase (CAT), glutathione peroxidase, glutathione reductase, and glucose-6-phosphate dehydrogenase [56]. Beyond melatonin itself, its metabolites, such as AFMK (N^1^-acetyl-N^2^-formyl-5-methoxykynuramine), may further reduce inflammatory responses and pain sensitivity. AFMK acts as a potent antioxidant that neutralizes free radicals and protects neurons from oxidative damage. By inhibiting lipid peroxidation, it further reduces oxidative stress-driven inflammation and may attenuate the sensitization of nociceptive pathways associated with chronic pain [52].

Based on the evidence discussed above, reduced melatonin secretion, a common feature of sleep disturbances, may promote neuroinflammation and increase susceptibility to pain sensitization [49].

In parallel with circadian rhythm and melatonin signaling, sleep deprivation is closely linked to dysregulation of the hypothalamic–pituitary–adrenal (HPA) axis. Chronic sleep disruption can lead to abnormal cortisol secretion patterns, impairing stress adaptation and promoting a pro-inflammatory state [9,14,50]. Elevated or dysregulated cortisol levels have been associated with heightened pain sensitivity, poorer sleep quality, and increased vulnerability to mood disorders. An experimental study further demonstrated that a single night of total sleep deprivation amplified both baseline cortisol levels and the cortisol response to a psychosocial stressor in healthy adults, indicating heightened HPA axis reactivity. In contrast, salivary alpha-amylase—a marker of sympathetic activation—rose in response to the stressor but was unaffected by sleep loss, suggesting selective sensitization of the slower-responding HPA axis. These findings indicate that sleep deprivation may promote allostatic load through sustained cortisol excess, potentially increasing vulnerability to stress-related health consequences [57].

Collectively, disturbances in circadian gene expression, melatonin signaling, and HPA axis regulation represent key molecular mechanisms linking sleep disruption to neuroinflammation and pain sensitization, thereby contributing to the reciprocal relationship between sleep disturbances and chronic pain [50].

### 4.4. Bidirectional Feedback Between Pain Intensity and Sleep Quality

The interaction between sleep and chronic pain is inherently bidirectional and multifactorial. As detailed above, sleep disturbances enhance pain sensitivity through disrupted N3 sleep, pro-inflammatory cytokine release (e.g., IL-6, TNF-α), and dysregulation of circadian clock genes, melatonin signaling, and the HPA axis. Conversely, persistent pain fragments sleep architecture, reducing total sleep time and selectively suppressing restorative N3 sleep, thereby perpetuating the very mechanisms that amplify nociception. This self-reinforcing cycle—in which worsening sleep quality and escalating pain intensity fuel one another—is further compounded by psychological comorbidities such as anxiety and depression, which are highly prevalent in chronic pain populations [36,37,38,39,45,48,49,50].

The convergence of these neural, neuroendocrine, and inflammatory mechanisms underscores the clinical importance of addressing sleep disturbances as a modifiable therapeutic target capable of mitigating pain sensitization and disrupting the reciprocal cycle between chronic pain and impaired sleep.

## 5. Shared Neurobiological Mechanisms of Psychological Comorbidities and Emotional Dysregulation

### 5.1. Clinical Interconnections Between Chronic Pain and Depression and Anxiety

The co-occurrence of chronic pain with anxiety and depression is a significant and prevalent clinical challenge [58]. Chronic pain affects multiple dimensions of well-being, contributing not only to physical suffering but also to emotional strain, diminished quality of life, and disruptions in social functioning [59]. Recent evidence indicates that a substantial proportion of the chronic pain population experiences clinically significant symptoms of depression or anxiety [58,60]. This relationship is particularly well documented in older adults, where the coexistence of chronic pain and depression is associated with greater functional decline and more complex treatment needs [61]. However, high rates of comorbid anxiety and depression are also observed in other demographic groups, especially younger adults, females, and individuals with nociplastic pain [58].

Clinical evidence shows that anxiety and depression are approximately five times more prevalent among individuals with chronic pain than in those without, representing a substantial portion of adults with persistent symptoms of anxiety and depression [60,62]. Moreover, patients with greater pain-related disability experience more severe depressive symptoms and poorer sleep quality, with sleep disturbances mediating the relationship between pain and mood disorders [1].

### 5.2. Monoaminergic Dysregulation Linking Pain, Sleep, and Affective Dysfunction

Chronic pain and persistent stress are associated with dysregulation of serotonergic, dopaminergic, and noradrenergic neurotransmission, systems that collectively regulate pain processing, emotional function, and sleep architecture [14,15,16,17].

Consistent with this, Humo et al. demonstrated in experimental rodent models that chronic pain-induced depressive behaviors are accompanied by reduced serotonin levels and receptor dysfunction across key brain regions such as the hippocampus and spinal cord, with antidepressant treatments such as amitriptyline or fluoxetine partially restoring serotonergic tone and alleviating pain-related affective symptoms [15]. Complementing these findings, Duo et al. demonstrated in experimental rodent models that neuropathic pain enhances the activity of dorsal raphe 5-HT neurons, leading to increased wakefulness and reduced non-REM sleep in mouse models, effects that can be reversed by serotonergic agents such as mirtazapine or selective 5-HT2A antagonists [16,17].

The clinical overlap between chronic pain and depression is reflected in the therapeutic use of antidepressants that enhance serotonergic and noradrenergic neurotransmission. In particular, SNRIs such as duloxetine have demonstrated efficacy in both depressive symptoms and pain, supporting the relevance of shared monoaminergic pathways to their therapeutic effects. In patients with major depressive disorder accompanied by clinically significant pain, duloxetine at 60 mg/day significantly improved both depressive symptoms and pain compared with placebo [63,64].

TCAs such as amitriptyline are also widely used for pain, often at lower doses than those traditionally employed for depression; clinical studies have demonstrated analgesic effects with doses around 25 mg/day, although efficacy may vary across pain conditions. Thus, while pharmacological targeting of serotonergic and noradrenergic systems provides a common therapeutic framework for chronic pain and depression, the optimal dose and treatment strategy may differ according to the indication and clinical context [65].

**Figure 6:**
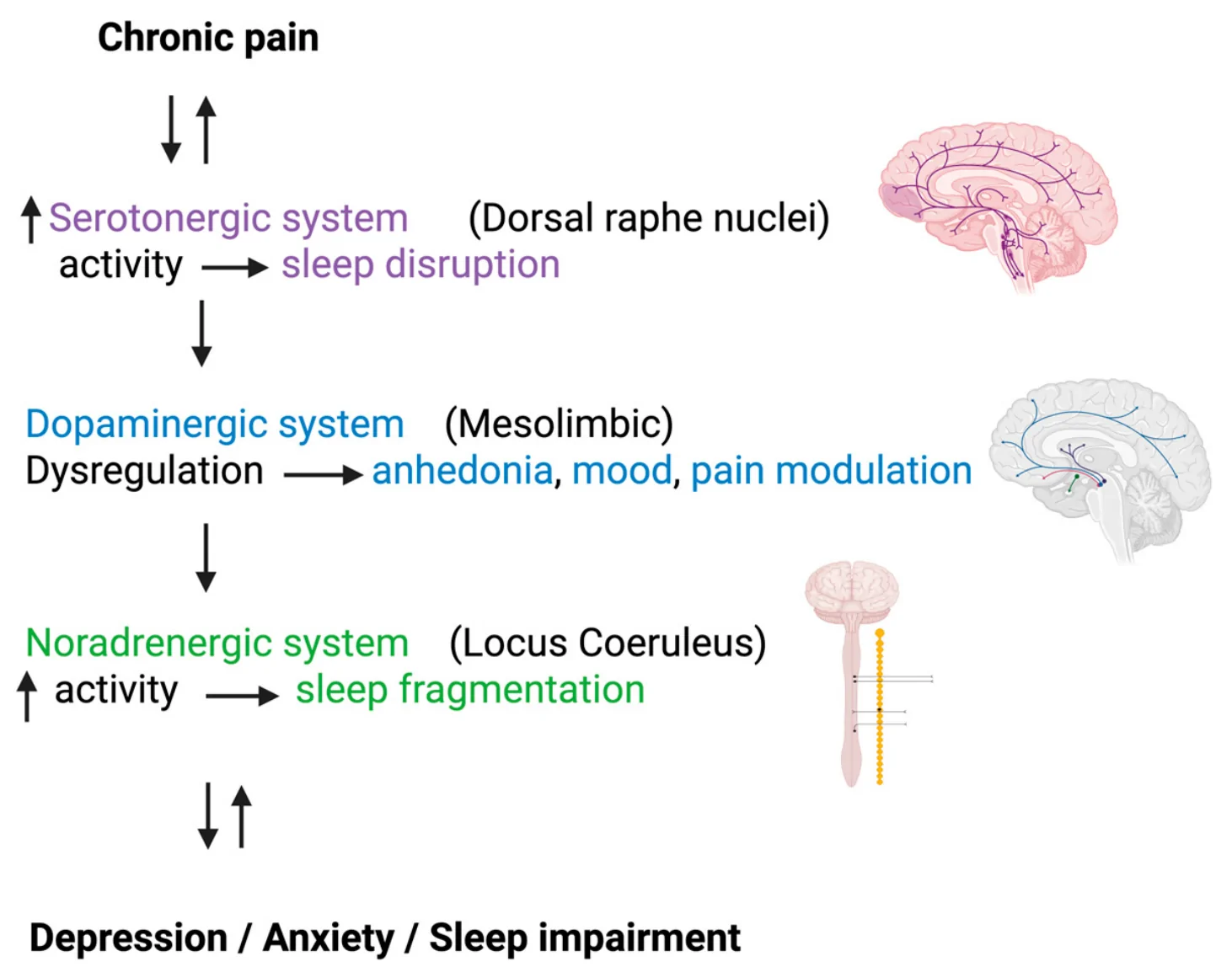
Schematic representation of monoaminergic neurotransmitter dysregulation linking chronic pain, sleep disturbances, and affective disorders. Persistent nociceptive signaling and chronic stress disrupt serotonergic, dopaminergic, and noradrenergic pathways involved in pain modulation, emotional regulation, and sleep architecture. Altered serotonergic signaling within the dorsal raphe nuclei contributes to sleep disruption and emotional dysregulation, while abnormal serotonergic activity may further reinforce chronic pain states. Dysregulation of the mesolimbic dopaminergic system, particularly within the nucleus accumbens and prefrontal cortex, impairs reward processing and pain modulation, contributing to anhedonia, depressive symptoms, and amplification of pain perception. Concurrently, increased noradrenergic activity originating from the locus coeruleus promotes hyperarousal and sleep fragmentation, further exacerbating pain sensitivity and affective symptoms. Through reciprocal interactions, these alterations establish a self-reinforcing neurobiological network that links chronic pain with depression, anxiety, and sleep impairment. Created in BioRender. Knezevic, N. (2026). https://BioRender.com/e66a7h3.

Parallel to serotonergic circuits, the dopaminergic system plays a pivotal role in the comorbidity of chronic pain, sleep disturbances, and mood disorders (Figure 6). Dopamine acts as both a neurotransmitter and neuromodulator capable of exerting inhibitory or excitatory effects, and clinical data indicate that over one-third of migraine patients exhibit dopaminergic symptoms such as yawning, somnolence, and nausea, highlighting the need for cautious modulation of this pathway. Experimental evidence from animal models suggests that the mesolimbic dopaminergic system serves as a key interface linking pain, insomnia, and depression. Pharmacological manipulation through partial agonism of D1 receptors and antagonism of D2 receptors produces both analgesic and hypnotic effects in neuropathic pain models [16]. Additionally, chronic pain and depression are associated with disrupted mesolimbic dopaminergic signaling within the nucleus accumbens and prefrontal cortex, contributing to impaired reward responsiveness, anhedonia, emotional dysregulation, and amplification of pain perception [17]. Reduced dopamine levels and receptor expression within the nucleus accumbens and prefrontal cortex contribute to a hypodopaminergic state, blunting reward processing and amplifying pain perception [15,16].

Complementing this dopaminergic dysregulation, the noradrenergic system, primarily originating from the locus coeruleus, serves as a fundamental inhibitory pathway for pain modulation. Preclinical research indicates that chronic pain and prolonged stress can significantly disrupt noradrenergic signaling, leading to time-dependent changes in tyrosine hydroxylase expression, receptor responsiveness, and norepinephrine transporter activity, alterations that coincide with the development of anxiety and depression like behaviors [15]. Experimental induction of sleep deprivation increased the levels of norepinephrine transporter mRNA in the locus coeruleus of rodents and of norepinephrine in the blood circulation in rodents and humans [14]. These elevations are consistent with heightened locus coeruleus activity, which has been linked to increased arousal and sleep fragmentation [15,16].

Overall, disruption of monoaminergic signaling represents a common neurobiological mechanism linking chronic pain, sleep disturbances, and affective dysfunction, contributing to the persistence and mutual reinforcement of these conditions [15,16,17].

### 5.3. Limbic Neuroplasticity and Neuroendocrine Dysregulation

Structural changes in the limbic system provide a parallel mechanism linking chronic pain to depression. Consistent with this, a clinical study of non-demented adults over the age of 70 years demonstrated that the effect of chronic pain on depressive symptoms is at least partially mediated by alterations in hippocampal volume, with a potentially specialized role for the right hippocampus. While a smaller right hippocampal volume is associated with both chronic pain and higher depressive symptoms, statistical path analysis reveals that the direct effect of chronic pain on depression is stronger than its indirect effect mediated through the hippocampus. Hippocampal atrophy represents one significant pathway, but chronic pain likely influences depression through a more complex neural network beyond this single structure [17].

Beyond neurotransmitter and structural alterations, another essential mechanism linking chronic pain with mood and sleep disturbances involves dysregulation of the body’s primary stress-response system, the hypothalamic pituitary adrenal (HPA) axis. The hypothalamic–pituitary–adrenal (HPA) axis plays a crucial role in regulating stress responses and is frequently dysregulated in chronic pain syndromes. Prolonged activation of this system can lead to abnormal cortisol secretion, resulting in either hypercortisolism or hypocortisolism. Interestingly, hypocortisolism often develops after a sustained period of hypercortisolism, reflecting the body’s reduced capacity to maintain high stress output over time. These hormonal alterations are associated with heightened pain sensitivity, lower pain thresholds, and impaired stress regulation. In addition, corticotropin-releasing hormone (CRH) and its receptors play a key role in stress-induced hyperalgesia, further linking neuroendocrine stress pathways with pain amplification [66]. Individuals with chronic pain become more vulnerable to anxiety and depression, which maintain and worsen the pain–stress–coping cycle [15,66].

## 6. Integrated Neuroimmune and Endocrine Mechanisms

### 6.1. Neuroimmune Signaling Networks and Glial Activation

Building upon the glial and neuroplastic mechanisms involved in central sensitization, chronic pain, sleep disturbances, and affective disorders are increasingly understood as manifestations of an interconnected neuroimmune signaling network rather than isolated pathological processes. Persistent nociceptive input and chronic psychological stress sustain activation of microglia and astrocytes within key pain- and emotion-processing regions of the central nervous system, including the spinal dorsal horn, limbic system, hippocampus, and hypothalamus. Astrocytes and microglia play complementary and interconnected roles in the development and persistence of chronic pain through activation of distinct but converging intracellular signaling pathways. Astrocytes contribute to chronic pain maintenance through reactive astrogliosis mediated by signaling cascades including JAK–STAT3, NF-κB, Notch–Olig2–Wnt, and TGF-β/Smad, whereas persistent microglial activation is characterized by microgliosis and induction of pathways involving CSF1–CSF1R, P2X4, p38 MAPK, and IRF8/IRF5 [27,28,29,30,67].

Rather than acting as isolated molecular processes, these pathways interact within a self-amplifying feed-forward inflammatory network that promotes sustained cytokine release, perpetuates central sensitization, and contributes to sleep disturbances, emotional dysregulation, and affective dysfunction [28,29,30,31].

### 6.2. Cytokine Signaling, Neuroinflammation, and Emotional Dysregulation

Elevated circulating levels of pro-inflammatory cytokines, including IL-1β, IL-6, IL-8, and TNF-α, contribute to a shared neuroinflammatory milieu linking chronic pain, sleep disturbances, and affective disorders through interconnected effects on neuroimmune signaling, neurotransmitter regulation, oxidative stress, and HPA-axis dysfunction [54,55]. NF-κB signaling serves as a major transcriptional regulator of this inflammatory response, promoting sustained cytokine production from activated glial cells and perpetuating neuroinflammatory signaling within pain- and emotion-processing pathways [29].

Within this inflammatory network, IL-1β and IL-6 are particularly implicated in impaired sleep continuity, altered sleep architecture, reduced synaptic plasticity, and development of depression-like behaviors [68,69]. Elevated IL-1β levels correlate with insomnia severity and are associated with increased NREM and reduced REM sleep, partially through NF-κB-mediated circadian disruption. In parallel, IL-1β suppresses BDNF signaling, thereby impairing neurogenesis and monoaminergic neurotransmission, while reductions in IL-1β levels have been associated with improvements in both depressive symptoms and sleep quality [68]. Similarly, IL-6 contributes to this comorbidity through its effects on neuroinflammation, neurotransmitter dysregulation, and HPA-axis activation. Elevated IL-6 levels are consistently associated with greater depressive symptom burden and impaired sleep continuity, while experimental studies demonstrate that increased IL-6 can induce depression-like behaviors that are reversible following its inhibition [70,71]. Elevated IL-6 concentrations have additionally been linked to reduced responsiveness to selective serotonin reuptake inhibitor (SSRI) therapy, suggesting a role in both disease severity and treatment resistance [72].

IL-8 and TNF-α further amplify this pathological state through sympathetic activation, oxidative stress, glial-mediated inflammatory signaling, and disruption of serotonergic neurotransmission [73,74,75]. Increased IL-8 levels have been observed in patients with depression and insomnia and correlate with symptom severity and treatment response, with potential sex-specific differences. Sex-related differences may influence several mechanisms linking chronic pain, sleep disturbances, and affective disorders. Preclinical evidence demonstrates sex-dependent mechanisms of persistent pain hypersensitivity, including differential involvement of spinal microglia, CGRP, and prolactin signaling [76]. Similarly, a recent meta-analysis found significantly elevated CRP and IL-6 levels in females with depression compared with healthy female controls, whereas corresponding differences were not observed in males; in contrast, the association between TNF-α and depression did not significantly differ by sex [77]. Sex may also modify the relationship between sleep disturbance and inflammation, although current findings remain mixed, highlighting the need for further sex-stratified research [78].

Chronic insomnia-related stress may further potentiate IL-8-driven inflammation via NF-κB activation and sympathetic dysregulation, reinforcing its potential role as both a biomarker and therapeutic target [73,74]. Elevated TNF-α levels are similarly associated with depressive severity and insomnia duration and mechanistically contribute through HPA-axis activation, reduced serotonin availability via indoleamine 2,3-dioxygenase (IDO) induction, and oxidative stress-mediated neuroinflammation [75].

### 6.3. Mitochondrial Dysfunction and Oxidative Stress

Glial-derived inflammatory mediators and oxidative stress additionally impair mitochondrial function and promote excessive mitochondrial reactive oxygen species (mtROS) production, further amplifying NF-κB- and MAPK-dependent inflammatory signaling and neuronal dysfunction [31,79]. Oxidative stress-induced mitochondrial dysfunction plays a central role in the pathophysiology of both chronic pain and depression by disrupting cellular energy metabolism and sustaining neuroinflammation. In chronic pain, mitochondrial impairment contributes to ATP depletion, neuronal hyperexcitability, and central sensitization, whereas in depression it impairs neuronal resilience and emotional processing within limbic structures such as the hippocampus [80,81].

Collectively, mitochondrial dysfunction and oxidative stress reinforce neuroinflammatory signaling and neuronal dysfunction, thereby contributing to the persistence of chronic pain, sleep disturbances, and affective symptoms.

### 6.4. HPA Axis Dysregulation and Neuroendocrine Amplification

Importantly, inflammatory signaling pathways closely interact with the hypothalamic–pituitary–adrenal (HPA) axis, which serves as a major interface between chronic stress, neuroinflammation, and neuroendocrine dysregulation. Persistent cytokine elevation and glial activation stimulate stress-response pathways and alter cortisol homeostasis, thereby impairing stress adaptation, promoting sleep fragmentation, and further amplifying inflammatory signaling [9,14,50]. Chronic dysregulation of cortisol secretion may impair normal circadian rhythmicity and reduce the anti-inflammatory regulatory capacity of the HPA axis, promoting sustained neuroimmune activation and increased neuronal vulnerability. Experimental evidence further suggests that sleep deprivation increases basal cortisol levels and enhances stress-induced cortisol responses, while the resulting glucocorticoid signaling may fail to adequately suppress pro-inflammatory cytokine production, particularly IL-6, reflecting impaired interaction between the HPA and immune systems and promoting persistent neuroinflammatory activity [14,57]. Moreover, prolonged exposure to abnormal glucocorticoid signaling has been associated with hippocampal dysfunction, altered emotional processing, and disturbances in monoaminergic neurotransmission, collectively contributing to the development and maintenance of chronic pain, depression, and sleep disorders [15,66].

## 7. Therapeutic Implications and Emerging Targets

### 7.1. Behavioral and Pharmacological Therapeutic Approaches

Among the therapeutic approaches discussed, behavioral interventions have the most established clinical evidence. Cognitive-behavioral and mindfulness-based interventions remain important non-pharmacological approaches for chronic pain conditions associated with sleep and mental health disturbances. Both cognitive behavioral therapy for insomnia (CBT-I) and CBT for pain (CBT-P) improve sleep parameters and pain outcomes in fibromyalgia; however, CBT-I appears to produce more durable improvements in sleep maintenance and sustained analgesic benefits [82]. Martínez et al. demonstrated that CBT-I showed significant superiority over sleep hygiene control, with 36.7% of the CBT-I group achieving clinically significant sleep efficiency post-treatment compared to only 18.5% in the control group. The CBT-I group also reported substantial psychological benefits, showing significant post-treatment reductions in anxiety, depression, and pain catastrophizing that were maintained at follow-up [83]. Complementing CBT approaches, mindfulness-based interventions may improve sleep quality indirectly through reduction of pain interference, anxiety, and depressive symptoms [84]. Clinical evidence also supports selected pharmacological approaches, with pregabalin plus paroxetine demonstrating superior improvement in pain and depressive symptom burden compared with other antidepressant combinations [85].

### 7.2. Cytokine-Targeted and Neuroimmune Therapies

In contrast to the clinically established behavioral approaches, cytokine-targeted strategies remain at an earlier and more heterogeneous stage of translational development. Beyond conventional pharmacological and behavioral interventions, emerging evidence suggests that modulation of neuroimmune and inflammatory pathways may represent a potential therapeutic avenue for the interconnected pathophysiology of chronic pain, depression, and sleep disturbances. As discussed previously, patients with depression and comorbid chronic insomnia frequently exhibit elevated circulating levels of pro-inflammatory cytokines, including TNF-α, IL-1β, and IL-6, accompanied by reduced concentrations of anti-inflammatory mediators such as IL-10, IL-13, and IL-4, reflecting a state of persistent neuroimmune dysregulation [86,87,88]. Specifically, reduced IL-10 signaling may contribute to persistent neuroinflammation and impaired suppression of pro-inflammatory cytokine production, thereby exacerbating depressive symptoms, sleep fragmentation, and neuroimmune dysregulation [88]. Notably, several antidepressant agents, including sertraline, clomipramine, and trazodone, may partially restore immune homeostasis through enhancement of IL-10-mediated anti-inflammatory activity [86,89]. Clinical evidence for cytokine-targeted therapies is currently context-specific rather than directly validated for the interconnected pain–sleep–anxiety/depression phenotype. For instance, dupilumab, a monoclonal antibody targeting the IL-4/IL-13 signaling axis, significantly improves sleep quality in patients with chronic rhinosinusitis with nasal polyps (CRSwNP), providing preliminary evidence that modulation of type 2 inflammatory signaling may influence sleep-related outcomes in inflammatory disease [89]. However, these findings are specific to the CRSwNP population, and whether IL-4/IL-13 pathway modulation has therapeutic utility for primary insomnia, chronic pain-related sleep disturbances, or the broader pain–sleep–mood interaction remains uncertain and requires further investigation. By contrast, the therapeutic relevance of IL-37 remains largely preclinical and mechanistic. IL-37, an anti-inflammatory cytokine within the IL-1 family, has been proposed as a potential modulator of depressive-like behaviors through suppression of excessive inflammatory and immune activation [90]. Experimental evidence has also suggested that IL-37 may influence hypothalamic–pituitary–adrenal (HPA) axis activity, potentially affecting cortisol regulation and processes relevant to sleep and emotional regulation [90]. However, these observations are based primarily on mechanistic and preliminary evidence, and the clinical relevance of IL-37 modulation as a therapeutic strategy for depression or sleep disturbances remains to be established. Further clinical and translational studies are therefore needed to determine whether targeting these neuroimmune pathways can produce meaningful benefits across the interconnected domains of chronic pain, sleep disturbance, and mood dysfunction.

### 7.3. Glial-Targeted Therapeutic Strategies

Building on the central role of reciprocal microglial–astrocytic interactions in sustaining neuroinflammation and central sensitization, targeting these glial pathways has emerged as a promising therapeutic strategy for chronic pain.

However, most glial-targeted strategies discussed below remain at the preclinical or experimental stage, with limited direct clinical validation for chronic pain.

Following nerve injury, activated microglia initiate neuroinflammation through release of IL-1β, TNF-α, BDNF, and other inflammatory mediators that promote central sensitization [27,28,30]. Several microglia-targeted therapies, including minocycline, CSF1R inhibitors, and NLRP3 inflammasome inhibitors, have shown potential in reducing microglial activation, neuroinflammatory signaling, and pain hypersensitivity. In particular, minocycline may additionally modulate BDNF-related neurotrophic pathways and neuronal excitability, while CSF1R inhibition suppresses microglial proliferation and microgliosis [91,92,93,94,95].

Astrocyte-targeted therapies aim to restore glutamate homeostasis, reduce neuroinflammation, and normalize aberrant glial signaling involved in chronic pain maintenance. Strategies such as EAAT2/GLT-1 upregulation reduce excessive synaptic glutamate accumulation and limit reactive astrogliosis, while connexin-43 gap junction inhibitors and CXCL1/CXCR2 antagonists suppress abnormal astrocytic communication, chemokine-driven sensitization, and neuronal hyperexcitability [96,97,98].

Broad-spectrum agents such as glucocorticoids (dexamethasone, prednisone, methylprednisolone) provide proof-of-concept for glial modulation, suppressing pro-inflammatory cytokine production, microglial activation, and astrocytic signaling within the CNS [99,100]. However, their long-term use is constrained by systemic toxicity and limited specificity. These central glial effects nonetheless underscore the therapeutic relevance of neuroimmune modulation and motivate the development of more selective, glial-targeted interventions capable of disrupting the self-reinforcing cycle of neuroinflammation and central sensitization.

Overall, the most comprehensive therapeutic approach likely emerges from integrating these various modalities. However, these strategies span different levels of evidence maturity, ranging from clinically established behavioral and pharmacological interventions to context-specific or early clinical cytokine-targeted approaches and predominantly preclinical glial-targeted strategies. Therapeutic strategies targeting inflammatory cytokines, microglial and astrocytic activation, neurotransmitter imbalance, and psychological dysfunction may therefore help address the shared neuroimmune and neuroplastic mechanisms underlying these interconnected disorders, although further clinical validation is required for many of the emerging neuroimmune and glial-targeted approaches.

## 8. Conclusions

Chronic pain, sleep disturbances, and anxiety and depressive disorders represent a highly interconnected triad driven by shared neurobiological, neuroimmune, and neuroendocrine mechanisms. Evidence from this review highlights the central role of peripheral and central sensitization, glial activation, and maladaptive neuroplasticity in sustaining chronic pain, while sleep disruption further exacerbates nociceptive sensitivity through inflammatory and circadian dysregulation. Concurrently, dysregulation of monoaminergic neurotransmission and limbic circuitry, together with HPA axis imbalance, contributes to the high prevalence of depression and anxiety in chronic pain populations, reinforcing a self-perpetuating cycle of pain, poor sleep, and emotional distress.

Understanding the reciprocal interactions among these disorders at the molecular, cellular, and systems levels may provide important insights into more effective therapeutic strategies. Recognition of these overlapping mechanisms may facilitate the development of integrated, mechanism-informed treatment approaches capable of targeting multiple dimensions of this interconnected triad simultaneously, rather than addressing pain, sleep impairment, and affective symptoms as isolated conditions.

Current therapeutic evidence further supports the importance of this integrated framework, as both non-pharmacological interventions, including cognitive-behavioral and mindfulness-based therapies, and pharmacological approaches can produce meaningful, yet frequently incomplete, improvements across pain, sleep, and psychological outcomes. In parallel, increasing emphasis on cytokine modulation and glial-targeted therapies represents a particularly compelling direction, as these strategies aim to disrupt the reciprocal interactions between inflammation, central sensitization, and sleep dysfunction.

Despite these advances, important challenges remain in fully understanding the complex interactions among chronic pain, sleep disturbances, and anxiety and depression disorders. Although substantial progress has been achieved through preclinical research, mechanistic evidence from human studies remains limited. This paucity of clinical evidence highlights a persistent translational gap between experimental findings and their clinical application. Furthermore, the available human studies remain heterogeneous with respect to patient populations, chronic pain conditions, outcome measures, and methodological approaches, limiting the generalizability and clinical applicability of current findings. Many glial-targeted and cytokine-modulating therapies have yet to undergo large-scale clinical validation, and their long-term efficacy and safety remain incompletely established.

Although broad-spectrum agents such as glucocorticoids provide proof-of-concept for targeting neuroinflammatory pathways, their systemic adverse effects underscore the need for more selective, cell-specific therapeutic strategies.

Future research should prioritize well-designed longitudinal and translational studies integrating molecular biomarkers, advanced neuroimaging, and objective sleep assessments to further elucidate the reciprocal mechanisms linking chronic pain, sleep disturbances, and anxiety and depression disorders while validating emerging therapeutic targets. Such efforts may facilitate the development of personalized, mechanism-based interventions targeting neuroimmune signaling, glial activation, circadian dysregulation, and mitochondrial dysfunction, ultimately improving outcomes across this interconnected clinical triad.

## Data Availability

No new data were created or analyzed in this study. Data sharing is not applicable to this article.
